# Validation of large language models (Llama 3 and ChatGPT-4o mini) for title and abstract screening in biomedical systematic reviews

**DOI:** 10.1017/rsm.2025.15

**Published:** 2025-03-24

**Authors:** Adriana López-Pineda, Rauf Nouni-García, Álvaro Carbonell-Soliva, Vicente F Gil-Guillén, Concepción Carratalá-Munuera, Fernando Borrás

**Affiliations:** 1 Network for Research on Chronicity, Primary Care and Health Promotion (RICAPPS), San Juan de Alicante, Spain; 2 Clinical Medicine Department, School of Medicine, Miguel Hernandez University, San Juan de Alicante, Spain; 3 Primary Care Research Center, Miguel Hernandez University, San Juan de Alicante, Spain; 4 Department of Statistics, Mathematics and Informatics, Miguel Hernandez University, San Juan de Alicante, Spain

**Keywords:** abstracting and indexing, artificial intelligence, machine learning, review literature as a topic

## Abstract

With the increasing volume of scientific literature, there is a need to streamline the screening process for titles and abstracts in systematic reviews, reduce the workload for reviewers, and minimize errors. This study validated artificial intelligence (AI) tools, specifically Llama 3 70B via Groq’s application programming interface (API) and ChatGPT-4o mini via OpenAI’s API, for automating this process in biomedical research. It compared these AI tools with human reviewers using 1,081 articles after duplicate removal. Each AI model was tested in three configurations to assess sensitivity, specificity, predictive values, and likelihood ratios. The Llama 3 model’s LLA_2 configuration achieved 77.5% sensitivity and 91.4% specificity, with 90.2% accuracy, a positive predictive value (PPV) of 44.3%, and a negative predictive value (NPV) of 97.9%. The ChatGPT-4o mini model’s CHAT_2 configuration showed 56.2% sensitivity, 95.1% specificity, 92.0% accuracy, a PPV of 50.6%, and an NPV of 96.1%. Both models demonstrated strong specificity, with CHAT_2 having higher overall accuracy. Despite these promising results, manual validation remains necessary to address false positives and negatives, ensuring that no important studies are overlooked. This study suggests that AI can significantly enhance efficiency and accuracy in systematic reviews, potentially revolutionizing not only biomedical research but also other fields requiring extensive literature reviews.

## Highlights

### What is already known

- Systematic reviews are essential for synthesizing research evidence, but the process is labor-intensive and time-consuming, particularly during the study selection phase. Traditionally, this selection is carried out manually by human reviewers to ensure thoroughness and quality.

### What is new

- This study validates the application of artificial intelligence (AI) models, specifically Llama 3 and ChatGPT-4o mini, in the screening of titles and abstracts for systematic reviews. The findings demonstrate that these AI models can enhance both efficiency and accuracy, achieving overall accuracy rates exceeding 90% and showing promising performance compared with manual review processes.

### Potential impact for RSM readers

- Integrating AI into systematic reviews could transform the management of large datasets by significantly reducing the time and effort required. These advancements suggest that AI can be a valuable complementary tool, offering improved automation without compromising quality, thereby optimizing the study selection process and potentially elevating the overall effectiveness of systematic reviews.

## 1 Introduction

A systematic review is a rigorous methodology used to synthesize research evidence on a specific topic, providing a comprehensive summary of the existing literature, identifying gaps, and informing clinical guidelines and policy-making. This process involves several key steps: defining a clear research question, developing a protocol, conducting an extensive literature search, screening and selecting relevant studies, extracting data, assessing the risk of bias, and synthesizing the findings both qualitatively and quantitatively.^1^ These steps are labor-intensive and time-consuming, often taking several months to years, particularly during the screening phase where numerous abstracts and full texts must be evaluated for eligibility.

Systematic literature reviews have played a crucial role in enabling researchers to remain current by consolidating the most robust evidence pertinent to their research inquiries. Given the rapidly increasing volume of published research papers, performing a systematic literature review manually has become increasingly challenging and impractical. As the number of publications continues to grow, the need for efficient and effective methods to manage this data deluge becomes more critical.^2^

Recent evidence indicates that artificial intelligence (AI) models, such as GPT-4, exhibit superior performance compared with human reviewers in several facets of academic review. These include comprehensibility, clarity of review, relevance of feedback, and accuracy of technical assessments.^3^ The integration of AI into systematic reviews has the potential to enhance accuracy, in addition to streamlining the review process. AI tools can assist in various stages, particularly in the literature search and screening phases, significantly reducing the time required for these tasks.^4^ For instance, AI can enhance the efficiency of study selection by automatically identifying and ranking relevant studies based on predefined criteria. This can enhance consistency and handle larger volumes of data more efficiently than human reviewers alone.^5^ Examples of AI tools used in systematic reviews include ASReview®,^6^ Rayyan®,^7^ Abstrackr®,^8^ Colandr®,^9^ and Covidence®,^10^ which have incorporated machine learning algorithms to facilitate the screening process. These tools can learn from reviewer decisions, prioritizing studies likely to meet inclusion criteria, thereby potentially speeding up the review process.

However, using AI in systematic reviews presents certain challenges. One significant concern is the potential for bias in AI algorithms, stemming from the training data or the inherent assumptions within the models used.^11^ Moreover, the “black box” nature of some AI algorithms raises transparency issues, making it difficult to understand and trust their decision-making processes.^12^ Despite these concerns, the advantages of AI in reducing the workload of systematic reviews are substantial, provided these tools are rigorously validated and appropriately applied.

The selection of articles following the initial search is a critical step in the systematic review process. This phase involves two primary stages: the screening of titles and abstracts, followed by the full-text review of potentially relevant studies. Traditionally, screening is conducted independently by two reviewers to minimize errors and bias, with discrepancies resolved through discussion or by consulting a third reviewer.^13^ This dual-review process ensures that all relevant studies are included and minimizes the risk of overlooking significant research. Tools like Rayyan and Covidence facilitate this process by providing platforms for reviewers to independently assess studies and resolve conflicts efficiently. Recently, these tools have integrated AI features to further support the screening process. For instance, Rayyan offers a semi-automated screening mode where AI predicts the relevance of studies based on reviewer inputs, potentially accelerating the screening phase.^7^

The implementation of AI in the screening phase underscores the importance of validating these tools to ensure their reliability and accuracy. Validation studies are essential to compare the performance of AI tools with traditional human review processes, which are considered the gold standard. By doing so, researchers can determine whether AI tools can achieve similar or better outcomes in terms of sensitivity and specificity in study selection.^14^ Ensuring the accuracy of AI in screening is crucial, as any missed studies can significantly affect the findings and conclusions of a systematic review. Thus, the objective of this study was to validate the use of large language model (LLM) tools, including Llama 3 and ChatGPT-4o mini, in the article selection process (title and abstract screening) of a biomedical systematic review.

## 2 Methods

This is a comparative validation study designed to evaluate the efficacy and accuracy of two AI tools in screening irrelevant titles and abstracts from a set of articles retrieved through a systematic review search. The manual review of titles and abstracts conducted by human reviewers was used as the reference standard (gold standard). The data collection occurred retrospectively after the manual screening had been completed. This study was conducted in an academic setting with standard technological resources, including access to the necessary platforms and cloud services for AI implementation.

The articles included in this study were those identified in a prior systematic review investigating the risk factors specific to postmenopausal women associated with the incidence of cardiovascular morbidity and mortality (PROSPERO reference: CRD42022323101). Potentially eligible articles (*n* = 1,397) were identified through searches conducted in the Medline, Embase, and Scopus databases in October 2022. The full search strategy is provided in Supplementary Material 1. After duplicate removal using the Rayyan platform, a total of 1,081 articles remained. The titles and abstracts of these articles were independently screened by human reviewers in the initial screening phase and were used for comparison with the AI-based screening tools.

The reference standard was the manual review: two human reviewers independently assessed titles and abstracts using Rayyan between November 22, 2022, and January 23, 2023, and a third reviewer resolved discrepancies. Articles were classified as “Excluded” if they did not meet the eligibility criteria outlined in Supplementary Material 2 and “Included” if they met the criteria or if there was insufficient information in the title and the abstract to make a definitive judgment, necessitating progression to the second phase of full-text review. The final inclusion/exclusion decisions from the manual screening served as the gold standard as it remains the validated method in systematic reviews for initial screening.

The AI tools (Index test) classified articles as “Included” or “Excluded” based on their training to detect patterns indicating irrelevant content in abstracts. The AI models did not have access to the results of the manual screening. The AI tools were employed in July 2024. The AI tools evaluated in the present study were the following.

### 2.1 Llama 3

In our research, we leveraged the computational capabilities of the Llama 3 70B language model, accessing it through the cloud infrastructure provided by Groq. This approach allowed us to harness the power of a state-of-the-art LLM while benefiting from Groq’s free-tier application programming interface (API) access, subject to certain usage limitations. The Llama 3 70B model is an LLM developed by Meta AI. It represents a significant advancement in natural language processing, with 70 billion parameters, allowing for complex language understanding and generation tasks. Groq, a technology company specializing in AI accelerators, offers cloud-based API access to various LLMs, including Llama 3 70B. Their infrastructure is designed to provide high-performance computing for AI workloads. Llama 3 was selected due to its completely free access through Groq, making it a viable alternative for researchers with limited budgets who require an advanced LLM without financial constraints. Additionally, as a non-OpenAI model, it allows us to assess how different LLM architectures perform in systematic review screening.

### 2.2 ChatGPT-4o mini

In our research, we employed the ChatGPT-4o mini language model, accessing it through OpenAI’s API. This methodological approach allowed us to leverage a sophisticated language model while maintaining cost-effectiveness, as OpenAI offers this service at a relatively low price point. We used OpenAI’s authentication protocols to securely access the API. Our queries were formatted according to OpenAI’s API specifications, ensuring optimal interaction with the model. Programmatic calls were made to OpenAI’s servers with Python, which processed our requests using the ChatGPT-4o mini model. The model’s outputs were received via the API and subsequently processed for analysis within our research framework. Compared with full GPT-4 models, ChatGPT-4o mini is offered at a reduced rate, making it accessible for research projects with limited budgets. OpenAI regularly updates its models, ensuring that we have access to the latest improvements in the ChatGPT architecture. The use of a standardized API ensures that other researchers can easily replicate our methodology. The approach demonstrates the potential for democratizing access to cutting-edge AI technologies in academic and scientific contexts. ChatGPT-4o mini was selected because it is an affordable and widely accessible version of GPT-4, allowing us to evaluate whether a lower-cost alternative maintains comparable performance to more expensive AI models. Given that ChatGPT-based models are emerging as the “gold standard” in systematic review screening, their inclusion ensures that our results are aligned with the state of the art in this field while remaining cost-effective.

The process carried out was the following:

1. API Integration: We interfaced with the Llama 3 70B model through Groq’s RESTful API, which allowed for seamless integration into our existing research pipeline, and similarly, ChatGPT-4o mini was integrated using OpenAI’s API in our setup.
2. Data Processing: Input data were preprocessed and formatted according to both Groq’s and OpenAI’s API specifications before being sent to the respective models.
3. Model Invocation: API calls were made to Groq’s servers for Llama 3 70B and to OpenAI’s servers for ChatGPT-4o mini, which in turn processed our requests using the corresponding models.
4. Response Handling: The models’ outputs were received via the APIs and subsequently post-processed for analysis.

While Groq offers free access to its API, it comes with certain constraints, similar to OpenAI’s API usage:

- Rate Limiting: There are restrictions on the number of API calls that can be made within a given time frame.
- Computational Resources: The free tier may have limitations on the amount of computational power available for each request.
- Model Availability: Access to specific models, including Llama 3 70B and ChatGPT-4o mini, may be subject to availability and potential queuing.

By using the Llama 3 70B model through Groq’s API and ChatGPT-4o mini through OpenAI’s API, we were able to conduct our research with cutting-edge language models without the need for extensive local computational resources. This approach demonstrates the potential for democratizing access to advanced AI models in scientific research, albeit with considerations regarding the limitations of free-tier services.

We used three different configurations for each AI model, as shown in Table 1. These configurations maintained the same system prompt and user prompt across all tests, ensuring that the overall task and context remained consistent. The system prompt sets the AI’s behavior or context, whereas the user prompt is the input that the model responds to directly. The key variation between the configurations was the temperature setting, which was adjusted to 0, 0.5, and 1. This adjustment allowed for varying levels of creativity and randomness in the AI’s responses, providing a comparison of how each model (Llama 3 70B and GPT-4o mini) performed under different conditions of deterministic versus exploratory output generation. The system and user prompts are detailed in Supplementary Material 3 and were based on the eligibility criteria of the systematic review.

**Table 1:** Configuration of the AI tools

AI tool	Model	System prompt	Temperature	User prompt
LLA_0	Llama 3 70B	system_prompt	0	user_prompt
LLA_1	Llama 3 70B	system_prompt	0.5	user_prompt
LLA_2	Llama 3 70B	system_prompt	1	user_prompt
GPT_0	GPT–4o mini	system_prompt	0	user_prompt
GPT_1	GPT–4o mini	system_prompt	0.5	user_prompt
GPT_2	GPT–4o mini	system_prompt	1	user_prompt

*Note:* Temperature = 0 means the model becomes more deterministic, meaning it will choose the highest probability word or token every time, resulting in more predictable and conservative responses. Temperature = 0.5 introduces some randomness but maintains a balance between creativity and coherence; the model will explore different options, but it still tends to follow the most probable responses. Temperature = 1 introduces more variability and creativity, allowing for more diverse and unexpected outputs; the model’s responses will be more exploratory and may feel less constrained. AI, artificial intelligence.

### 2.3 Statistical analysis

Sensitivity, specificity, positive predictive value (PPV), negative predictive value, and likelihood ratios were calculated for each AI tool compared with the human gold standard. These metrics were derived from 2 × 2 tables, with 95% confidence intervals calculated for each measure. No indeterminate results were reported as both AI tools and human reviewers classified articles as either included or excluded. All articles were fully screened by both the AI tools and the human reviewers; hence, there were no missing data. A sensitivity analysis was performed by adjusting different prompt configurations and parameters of the AI tools to assess potential variability in diagnostic accuracy.

## 3 Results

**Figure 1:**
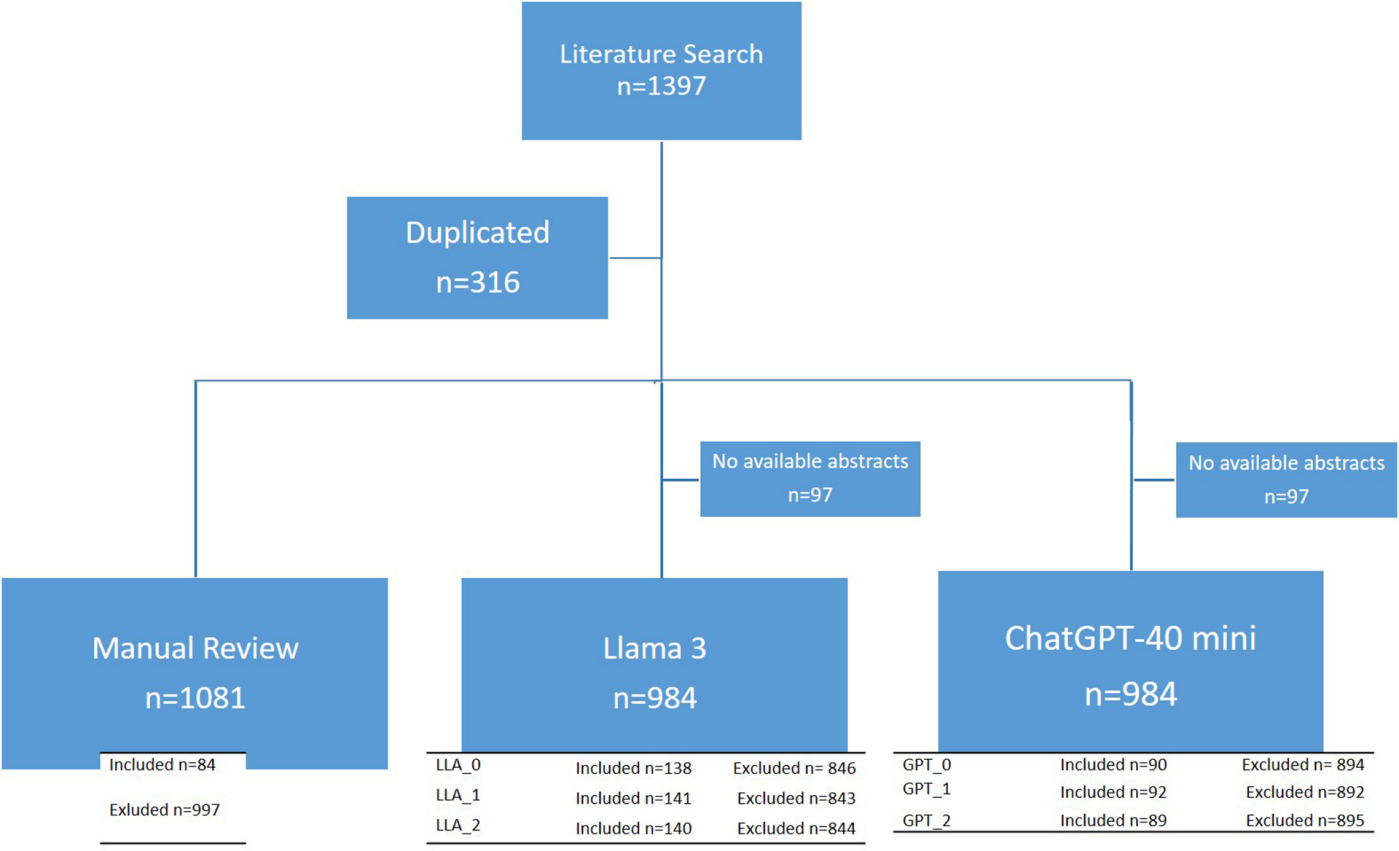
Flowchart of the screening process through manual review and artificial intelligence tools.

After conducting the literature search and removing duplicates, 1,081 articles advanced to the title and abstract screening phase of the systematic review. Of these, 84 studies were classified as “Included” by the researchers and proceeded to the second phase of full-text review. However, the reference files uploaded to Rayyan and the AI tools did not contain abstracts for 97 of the 1,081 retrieved articles. Consequently, human reviewers sought these abstracts through alternative platforms, but the AI tools could only assess the 984 abstracts available in the downloaded files. In this study, two AI tools—the Llama 3 model and ChatGPT-4o mini—were applied with three configurations each, as described in Table 1. The results of the AI classification, compared with the researchers’ classifications, are shown in Figure 1.

Tables 2 and 3 present the validity indicators. For the Llama 3 model, all three configurations displayed similar validity indicators, with LLA_2 demonstrating the best performance. Of the 984 studies, LLA_2 correctly included 62 of 80 (sensitivity: 77.5%); the AI correctly excluded 826 of 904 studies (specificity: 91.4%). LLA_2 incorrectly included 78 of 904 (false positives: 8.6%), and incorrectly excluded 18 of 80 (false negatives: 22.5%). The PPV and negative predictive value (NPV) were 44.3% and 97.9%, respectively, with positive and negative likelihood ratios of 9.0 and 0.2, respectively. LLA_2 achieved an overall accuracy rate of 90.2%.

**Table 2:** Validity indicators for each AI configuration

AI	VP	VN	FP	FN	TA	S	IC95	E	IC95
LLA_0	60	826	78	20	0.900	0.750	(0.655–0.845)	0.914	(0.896–0.932)
LLA_1	60	823	81	20	0.897	0.750	(0.655–0.845)	0.910	(0.891–0.929)
LLA_2	62	826	78	18	0.902	0.775	(0.683–0.867)	0.914	(0.896–0.932)
GPT_0	45	859	45	35	0.919	0.562	(0.453–0.671)	0.950	(0.936–0.964)
GPT_1	45	857	47	35	0.917	0.562	(0.453–0.671)	0.948	(0.934–0.962)
GPT_2	45	860	44	35	0.920	0.562	(0.453–0.671)	0.951	(0.937–0.965)

AI, artificial intelligence.

**Table 3:** Validity indicators for each AI configuration (continuation)

AI	VPP	IC95	VPN	IC95	LRP	IC95	LRN	IC95
LLA_0	0.435	(0.352–0.518)	0.976	(0.966–0.986)	8.721	(6.812–11.165)	0.274	(0.187–0.401)
LLA_1	0.426	(0.344–0.508)	0.976	(0.966–0.986)	8.333	(6.534–10.628)	0.275	(0.188–0.402)
LLA_2	0.443	(0.361–0.525)	0.979	(0.969–0.989)	9.012	(7.069–11.489)	0.246	(0.164–0.370)
GPT_0	0.500	(0.397–0.603)	0.961	(0.948–0.974)	11.240	(7.967–15.858)	0.461	(0.359–0.591)
GPT_1	0.489	(0.387–0.591)	0.961	(0.948–0.974)	10.808	(7.701–15.169)	0.462	(0.36–0.593)
GPT_2	0.506	(0.402–0.61)	0.961	(0.948–0.974)	11.469	(8.106–16.227)	0.461	(0.359–0.591)

*Note:* VP, true positive; VN, true negative; FP, false positive; FN, false negative; TA, total accuracy; S, Sensitivity; E, specificity; PPV, positive predictive value; NPV, negative predictive value; LR+, positive likelihood ratio; LR−, negative likelihood ratio; 95% CI, 95% confidence interval; AI, artificial intelligence.

For the ChatGPT-4o mini model, the three configurations also displayed similar validity indicators, with CHAT_2 showing the best performance. Of the 984 studies, CHAT_2 correctly included 45 of 80 (sensitivity: 56.2%) and correctly excluded 860 of 904 (specificity: 95.1%). CHAT_2 incorrectly included 44 of 904 (false positives: 4.9%) and incorrectly excluded 35 of 80 (false negatives: 43.7%). The PPV and the NPV were 50.6% and 96.1%, respectively, with positive and negative likelihood ratios of 11.5 and 0.5, respectively. CHAT_2 achieved an overall accuracy rate of 92.0%.

## 4 Discussion

This study demonstrates that the use of AI tools, such as the Llama 3 model and ChatGPT-4o mini, for screening titles and abstracts in systematic reviews within the biomedical field is valid, achieving acceptable sensitivity, high specificity, and an overall accuracy rate of 90%.

Two previous studies^15^ ^,^ ^16^ compared the effectiveness of AI tools in selecting titles and abstracts for systematic reviews with human manual review. One study evaluated three machine learning software tools (Rayyan, Abstrackr, and Colandr), which were found to be useful in accelerating the title selection process, with Rayyan being the most effective among them, showing results similar to those of our study (sensitivity: 78% vs. 77.5%; specificity: 99% vs. 91%). The other study focused on assessing specific machine learning methods, observing that these tools can significantly reduce workload and human error, though they remain susceptible to errors in classifying relevant and irrelevant articles. A recent study evaluated language models (LLMs) in the selection of titles and abstracts for systematic reviews, showing variations in performance depending on the dataset used. In the 10 published datasets, the classifiers demonstrated high sensitivity but low specificity: FlanT5 (94.48%/31.78%), OpenHermes-NeuralChat (97.58%/19.12%), Mixtral (81.93%/75.19%), and Platypus 2 (97.58%/38.34%). In a manually created dataset, all models achieved 100% sensitivity but with much lower specificity: FlanT5 (12.58%), OpenHermes-NeuralChat (4.54%), Mixtral (62.47%), and Platypus 2 (24.74%). The results indicate that small adjustments in configurations can significantly impact performance, highlighting the importance of customization in the classification of scientific publications.^17^

Regarding the use of ChatGPT, our sensitivity and specificity results for title and abstract selection were consistent with previous studies,^1^ ^,^ ^18^ which reported similar specificity parameters (90%–93% vs. 95%). However, the sensitivity results were not as closely aligned (67%–84% vs. 56.2%). Both studies concluded that, despite ChatGPT-4’s limitations in systematic reviews, it shows promising potential for study selection. With appropriate adjustments, its integration could enhance the efficiency and accuracy of the process, providing new opportunities to automate and optimize systematic reviews.^18^ ^,^ ^19^

Recently published studies have further supported these findings. One study evaluating GPT-3.5 Turbo as a sole reviewer for systematic reviews demonstrated variable performance depending on the rules applied. With a balanced rule, sensitivity ranged from 81.1% to 96.5%, while specificity ranged from 25.8% to 80.4%, with GPT identifying 1% of relevant citations missed by humans, at the cost of 45.3% false positives. When applying a more sensitive rule, sensitivity increased to 94.6%–99.8%, but specificity decreased to 2.2%–46.6%. Despite these trade-offs, the study highlighted GPT-3.5 Turbo’s potential to support systematic reviews, reducing manual work but at the risk of missing a small percentage of relevant citations.^20^

Another study focused on a three-level selection method using GPT-3.5 and GPT-4 in systematic reviews showed promising results. GPT-4 outperformed GPT-3.5 in specificity (99.6% vs. 70.9%), with comparable sensitivity (80.6% vs. 90.0%). The models accurately included records used for the meta-analyses in both reviews (six and nine records, respectively). After adjusting for justifiable exclusions, GPT-4’s final sensitivity and specificity reached 96.2%/99.6% and 94.3%/85.5% in the two studies. While GPT-3.5 faced issues with incorrect exclusions due to a lack of domain knowledge, GPT-4 showed consistent performance, supporting its practical use in systematic reviews and emphasizing the need to generalize its application across various settings.^21^

Our findings align with previous research highlighting the potential of AI tools to reduce workload in systematic reviews. For instance, Yao et al.^22^ found that tools like Abstrackr and EPPI-Reviewer offer significant time savings without compromising the inclusion of relevant studies, similar to the efficiency observed with Llama 3 and ChatGPT-4o mini in our study. O’Mara-Eves et al.^11^ emphasized that text mining can reduce workload by 30%–70%, but cautioned against the possible omission of a small percentage of relevant studies, a risk mitigated in our analysis due to the high sensitivity of the Llama 3 model. Additionally, Valizadeh et al.^23^ evaluated the Rayyan tool, illustrating how threshold settings can impact accuracy and specificity, a factor we explored by adjusting AI parameters in our study to optimize the balance between sensitivity and specificity. Although Van Dijk et al.^14^ demonstrated that tools like ASReview are promising when used correctly, accelerating the selection process, they did not assess the model’s accuracy or validity indicators, which are central to our research. This underscores the necessity of carefully tuning and validating AI models to ensure methodological quality at each stage of the review process. Combining time efficiency with rigorous accuracy and validity assessment is crucial to ensure that automation does not compromise the quality of the review.

Our study expands upon this prior work by explicitly reporting the optimized prompts and configurations used, allowing researchers with programming knowledge to easily replicate and adapt these methods. This transparency ensures that these tools can be employed effectively for screening titles and abstracts in any systematic review. Moreover, we demonstrate that these approaches can be extended to more advanced AI models in the future, potentially achieving even greater sensitivity and specificity. However, we recognize that many researchers conducting systematic reviews may not have programming expertise. To address this, we have designed our approach to be as accessible as possible by detailing the necessary prompts and configurations. Future developments could further improve usability by integrating these models into existing platforms such as Covidence or Rayyan or by creating user-friendly interfaces that allow researchers to apply AI tools without requiring direct API interaction.

In the context of this study, the false negative rate (complement of sensitivity) indicates the articles that the AI might miss, potentially leading to the loss of important studies. The false positive rate (complement of specificity) represents articles that, despite exclusion criteria, would be unnecessarily analyzed. Probability coefficients according to Evidence-Based Medicine^24^ are used in decision-making and indicate that changes from pre-test to post-test probability can be very important, important, moderate, small, or of no clinical significance. Therefore, in decision-making when using AI for systematic reviews, the changes generated when an article is included are moderate to high if included and moderate if excluded. Probability coefficients are tools used in medicine to interpret and make decisions based on signs and symptoms of a disease.^24^ In this review, within the context of article selection using AI compared with a human manual review (considered the gold standard), probability coefficients are used to evaluate AI effectiveness in identifying articles manually included in the title and abstract screening process.

Regarding validity indicators, the Llama 3 model (LLA_2 configuration) achieved the best results in this study. Analyzing the NPV, this model demonstrates high efficacy in excluding irrelevant articles, as indicated by its high NPV, ranging from 97.6% to 97.9%, ensuring that articles discarded by the model largely align with those excluded by human reviewers. However, the PPV is moderate, ranging from 42.6% to 44.3%, due to the low prevalence of relevant articles (8.1%). Although the model improves the probability of identifying relevant articles, the PPV is constrained by the limited number of these in the sample, reflecting the inherent challenge of selecting relevant articles in a low-prevalence context, with PPV being significantly influenced by this aspect.

For the ChatGPT model, results show balanced performance but less precision in excluding irrelevant articles, with an NPV of 96.1%, indicating slightly less alignment with human reviewers compared with Llama 3. However, ChatGPT offers a slightly higher PPV, ranging from 48.9% to 50.6%, suggesting a better ability to identify relevant articles, although this PPV is also influenced by the low prevalence of 8.1%. Overall, ChatGPT shows adequate performance, with a higher PPV than Llama 3 but slightly reduced exclusion precision.

By integrating both cost-free options (e.g., Llama 3 via Groq) and accessible variants (e.g., ChatGPT-4o mini), this study provides a practical framework for researchers with limited resources to leverage state-of-the-art AI tools for systematic review screening. Additionally, by simplifying implementation through standardized scripts and templates, we aim to lower technical barriers and facilitate adoption by a broader audience. The detailed prompts and configurations reported allow researchers to set up workflows efficiently, ensuring replicability and reducing entry barriers to using AI in this context.

Regarding study limitations, reliance on AI algorithms is notable, as the effectiveness of these tools heavily depends on the quality of the algorithm and training data. If the model has not been trained on a sufficiently representative sample of biomedical articles, it may not generalize well to other datasets, although we consider the sample to be representative in this case. Additionally, as previously mentioned, there is a risk that the AI might miss some relevant articles or include studies not manually selected. This could increase workload by necessitating a full-text review of articles that may not be genuinely relevant, leading to a second phase of the study to assess the suitability of AI-included and excluded studies compared with manual review. There are also inherent limitations in human reviewer training. Although AI has shown efficacy, human intervention remains necessary to validate and verify exclusion and inclusion decisions, potentially limiting time savings in practical settings. These limitations highlight the importance of using AI as a complementary tool rather than a complete replacement for manual review in systematic reviews.

In this study, Llama 3 (LLA_2) identified 140 articles for full review compared with 84 manually selected, with 62 overlaps. The AI suggested 78 additional articles and excluded 22 articles that human reviewers would have included. It is crucial to determine if those 22 articles excluded by the AI should have been retained and whether the AI correctly identified relevant articles overlooked in the manual review. A second phase of the study would assess the suitability of articles selected by AI (false positives) and those excluded by AI but included manually (false negatives). This phase would evaluate whether these articles progress to the final stage of the systematic review after a more thorough full-text review or if the manual review of the false positives and false negatives would suffice, saving time. This analysis would determine whether the articles identified or excluded by AI significantly impact the final review results. Comparing results with and without these articles would assess AI effectiveness and its ability to identify key studies missed in the manual review. This approach would validate the utility of AI in systematic reviews, which is essential given the increasing volume of scientific publications.^25^ AI enables automation of article search and selection, reducing human errors and improving efficiency and accuracy, adapting to current research needs.^3^ AI has demonstrated efficiency in the title and abstract screening phase, enhancing process quality by complementing and, in some cases, surpassing manual review in identifying relevant studies. It is recommended to use AI to exclude irrelevant articles, with additional human review of AI-included articles to ensure a more accurate full-text evaluation. With the increasing volume of scientific literature, AI presents promising potential to optimize systematic reviews across different contexts, including biomedical research. Several studies have validated models such as GPT-3.5, GPT-4, Llama 3 70B, and ChatGPT-4o mini, demonstrating complementary capabilities to reduce human workload and minimize errors in the selection of titles and abstracts. Although the evaluated models have shown strong specificity and accuracy, their sensitivity and the need for manual validation highlight areas for improvement. These results emphasize the need for continued research and adaptation of these tools, adjusting selection criteria to address the particularities of other disciplines, and validating their performance before widespread adoption.^20^ ^,^ ^21^ AI has the potential to revolutionize the efficiency and accuracy of systematic reviews, expanding its applicability beyond the biomedical field to other domains requiring extensive literature reviews.

## 5 Conclusion

This study shows that AI, specifically the Llama 3 model, is a valid, efficient, and accurate tool for study selection in the process of screening titles and abstracts for a biomedical systematic review. Our results indicate that while AI can significantly reduce workload by identifying and excluding irrelevant articles, it is crucial to complement its use with manual review to ensure that no relevant studies are omitted. Combining AI with human review optimizes the selection process, maintaining high methodological standards and contributing to the final quality of the systematic review. Further research is needed to assess its ability to evaluate the suitability of selected articles and its impact on final results. This will promote greater confidence in the adoption of these tools in scientific practice, enabling systematic reviews to be faster, more accurate, and sustainable in the context of the growing volume of scientific literature.

## Supporting information

López-Pineda et al. supplementary material López-Pineda et al. supplementary material

## Data Availability

The data that support the findings of this study are available at https://docs.google.com/spreadsheets/d/1-4GIh1XytLks2ZMZ8kZM7MHIVPQ9qOj3/edit?usp=sharing&ouid=107391628292051032027&rtpof=true&sd=true.

## Supplementary material

To view supplementary material for this article, please visit http://doi.org/10.1017/rsm.2025.15.
