# Supplementary material for: Validation of large language models (Llama 3 and ChatGPT-4o mini) for title and abstract screening in biomedical systematic reviews
Source: Res Synth Methods. 2025 Mar 24;16(4):620–30. doi: 10.1017/rsm.2025.15 (PMC12623132; doi:10.1017/rsm.2025.15)
Supplement: Supplementary file 1 [file S1759287925000158sup001.zip › Supplementary_Material_1.docx]

**Supplementary Material 1.** Search strategies.

- **Search terms and keywords:**

The following set of MeSH (Medical Subject Headings) and keywords were combined to identify relevant studies:

(("cardiovascular diseases"[MeSH Terms] OR "cardiovascular diseases"[Title/Abstract] OR "inferior wall myocardial infarction"[MeSH Terms] OR "anterior wall myocardial infarction"[MeSH Terms] OR "angina pectoris"[MeSH Terms] OR "stroke"[MeSH Terms] OR "stroke"[Title/Abstract] OR "embolic stroke"[MeSH Terms])

AND ("menopause"[MeSH Terms] OR "menopaus*"[Title/Abstract] OR "postmenopause"[MeSH Terms] OR "premenopause"[MeSH Terms] OR "Climacteric"[Title/Abstract] OR "menopausal age"[Title/Abstract])

AND ("parturition"[MeSH Terms] OR "delivery, obstetric"[MeSH Terms] OR "Childbirth"[Title/Abstract]))

OR (("abortion, induced"[MeSH Terms] OR "abortion, spontaneous"[MeSH Terms] OR "Abortion"[Title/Abstract] OR "Pregnancy loss"[Title/Abstract] OR "Miscarriage"[Title/Abstract]))

AND ("hysterectomy"[MeSH Terms] OR "Hysterectomy"[Title/Abstract] OR "contraceptives, oral, hormonal"[MeSH Terms] OR "contraceptive devices"[MeSH Terms] OR "contraceptive agents"[MeSH Terms]))

- **Filters:**

The following filters were applied:

**Study type:**

- Classical Article
- Clinical Study
- Clinical Trials (Phase I - IV)
- Comparative Study
- Meta-Analysis
- Observational Study
- Randomized Controlled Trial
- Review

**Study Population:**

- Humans
- Sex: Female
- Age: Adult (19+ years)
- **Database:** PubMed
  - **Date of search:** June 30 2022
  - **Search strategy with filters:**

((("cardiovascular diseases"[MeSH Terms] OR "cardiovascular diseases" [Title/Abstract] OR "inferior wall myocardial infarction"[MeSH Terms] OR "anterior wall myocardial infarction"[MeSH Terms] OR "angina pectoris" [MeSH Terms] OR "stroke"[MeSH Terms] OR "stroke"[Title/Abstract] OR "embolic stroke"[MeSH Terms] OR ("Schemic"[All Fields] AND "stroke" [MeSH Terms]) OR "Angina"[Title/Abstract] OR "heart attack" [Title/Abstract] OR "acute myocardial infarction"[Title/Abstract] OR "coronary disease"[Title/Abstract]) AND ("menopause"[MeSH Terms] OR "menopaus*"[Title/Abstract] OR "postmenopause"[MeSH Terms] OR "premenopause"[MeSH Terms] OR "Climacteric"[Title/Abstract] OR "menopausal age"[Title/Abstract] OR "Age of menopause"[Title/Abstract] OR "Age at menopause"[Title/Abstract]) AND ("Parturition"[MeSH Terms] OR "delivery, obstetric"[MeSH Terms] OR "Parturition"[Title/Abstract] OR "delivery, obstetric"[MeSH Terms] OR "Delivery"[Title/Abstract] OR "Childbirth"[Title/Abstract] OR "Age at first childbirth"[Title/Abstract] OR "Birth"[Title/Abstract])) OR (("cardiovascular diseases"[MeSH Terms] OR "cardiovascular diseases"[Title/Abstract] OR "inferior wall myocardial infarction"[MeSH Terms] OR "anterior wall myocardial infarction"[MeSH Terms] OR "angina pectoris"[MeSH Terms] OR "stroke"[MeSH Terms] OR "stroke"[Title/Abstract] OR "embolic stroke"[MeSH Terms] OR ("Schemic" [All Fields] AND "stroke"[MeSH Terms]) OR "Angina"[Title/Abstract] OR "heart attack"[Title/Abstract] OR "acute myocardial infarction" [Title/Abstract] OR "coronary disease"[Title/Abstract]) AND ("menopause" [MeSH Terms] OR "menopaus*"[Title/Abstract] OR "postmenopause" [MeSH Terms] OR "premenopause"[MeSH Terms] OR "Climacteric" [Title/Abstract] OR "menopausal age"[Title/Abstract] OR "Age of menopause"[Title/Abstract] OR "Age at menopause"[Title/Abstract]) AND ("abortion, induced"[MeSH Terms] OR "abortion, spontaneous"[MeSH Terms] OR "Abortion"[Title/Abstract] OR "Pregnancy loss"[Title/Abstract] OR "Miscarriage"[Title/Abstract])) OR (("cardiovascular diseases"[MeSH Terms] OR "cardiovascular diseases"[Title/Abstract] OR "inferior wall myocardial infarction"[MeSH Terms] OR "anterior wall myocardial infarction"[MeSH Terms] OR "angina pectoris"[MeSH Terms] OR "stroke" [MeSH Terms] OR "stroke"[Title/Abstract] OR "embolic stroke"[MeSH Terms] OR ("Schemic"[All Fields] AND "stroke"[MeSH Terms]) OR "Angina" [Title/Abstract] OR "heart attack"[Title/Abstract] OR "acute myocardial infarction"[Title/Abstract] OR "coronary disease"[Title/Abstract]) AND ("menopause"[MeSH Terms] OR "menopaus*"[Title/Abstract] OR "postmenopause"[MeSH Terms] OR "premenopause"[MeSH Terms] OR "Climacteric"[Title/Abstract] OR "menopausal age"[Title/Abstract] OR "Age of menopause"[Title/Abstract] OR "Age at menopause"[Title/Abstract]) AND ("menstrual cycle"[MeSH Terms] OR "menstrual cycle" [Title/Abstract])) OR (("cardiovascular diseases"[MeSH Terms] OR "cardiovascular diseases"[Title/Abstract] OR "inferior wall myocardial infarction"[MeSH Terms] OR "anterior wall myocardial infarction"[MeSH Terms] OR "angina pectoris"[MeSH Terms] OR "stroke"[MeSH Terms] OR "stroke"[Title/Abstract] OR "embolic stroke"[MeSH Terms] OR ("Schemic" [All Fields] AND "stroke"[MeSH Terms]) OR "Angina"[Title/Abstract] OR "heart attack"[Title/Abstract] OR "acute myocardial infarction" [Title/Abstract] OR "coronary disease"[Title/Abstract]) AND ("menopause" [MeSH Terms] OR "menopaus*"[Title/Abstract] OR "postmenopause" [MeSH Terms] OR "premenopause"[MeSH Terms] OR "Climacteric" [Title/Abstract] OR "menopausal age"[Title/Abstract] OR "Age of menopause"[Title/Abstract] OR "Age at menopause"[Title/Abstract]) AND ("Hysterectomy"[MeSH Terms] OR "Hysterectomy"[Title/Abstract] OR "hysterectomy, vaginal"[MeSH Terms])) OR (("cardiovascular diseases" [MeSH Terms] OR "cardiovascular diseases"[Title/Abstract] OR "inferior wall myocardial infarction"[MeSH Terms] OR "anterior wall myocardial infarction"[MeSH Terms] OR "angina pectoris"[MeSH Terms] OR "stroke" [MeSH Terms] OR "stroke"[Title/Abstract] OR "embolic stroke"[MeSH Terms] OR ("Schemic"[All Fields] AND "stroke"[MeSH Terms]) OR "Angina" [Title/Abstract] OR "heart attack"[Title/Abstract] OR "acute myocardial infarction"[Title/Abstract] OR "coronary disease"[Title/Abstract]) AND ("menopause"[MeSH Terms] OR "menopaus*"[Title/Abstract] OR "postmenopause"[MeSH Terms] OR "premenopause"[MeSH Terms] OR "Climacteric"[Title/Abstract] OR "menopausal age"[Title/Abstract] OR "Age of menopause"[Title/Abstract] OR "Age at menopause"[Title/Abstract]) AND ("contraceptives, oral, hormonal"[MeSH Terms] OR "contraceptive agents"[MeSH Terms] OR "contraceptive agents, female"[MeSH Terms] OR "contraceptives, oral, hormonal"[MeSH Terms] OR "contraceptive devices" [MeSH Terms] OR "contraceptive devices, female"[MeSH Terms] OR "contraceptives, oral"[MeSH Terms] OR "Contracept"[Title/Abstract]))) AND ((classicalarticle[Filter] OR clinicalstudy[Filter] OR clinicaltrial[Filter] OR clinicaltrialphasei[Filter] OR clinicaltrialphaseii[Filter] OR clinicaltrialphaseiii[Filter] OR clinicaltrialphaseiv[Filter] OR comparativestudy[Filter] OR controlledclinicaltrial[Filter] OR evaluationstudy[Filter] OR meta-analysis[Filter] OR multicenterstudy[Filter] OR observationalstudy[Filter] OR preprint[Filter] OR randomizedcontrolledtrial[Filter] OR review[Filter]) AND (humans[Filter]) AND (female[Filter])

- **Results:** 244 documents
- **Database:** Scopus
  - **Date of search:** October, 14 2022
  - **Search strategy with filters:**

#1 ( TITLE-ABS-KEY ( "cardiovascular disease" ) OR TITLE-ABS-KEY ( "Myocardial Infarction" ) OR TITLE-ABS-KEY ( "Angina" ) OR TITLE-ABS-KEY ( "Stroke" ) OR TITLE-ABS-KEY ( "Heart attack" ) OR TITLE-ABS-KEY ( "Coronary disease" ) OR TITLE-ABS-KEY ( "Coronary disease" ) OR TITLE-ABS-KEY ( "Cardiovascular mortality" ) OR TITLE-ABS-KEY ( "Acute myocardial syndrome" ) ) AND ( TITLE-ABS-KEY ( menopause ) OR TITLE-ABS-KEY ( postmenopause ) OR TITLE-ABS-KEY ( premenopause ) OR TITLE-ABS-KEY ( "menopausal age" ) OR TITLE-ABS-KEY ( climacteric ) OR TITLE-ABS-KEY ( "Age of menopause" ) OR TITLE-ABS-KEY ( "Age at menopause" ) OR TITLE-ABS-KEY ( menopaus* ) ) AND ( TITLE-ABS-KEY ( parturition ) OR TITLE-ABS-KEY ( delivery ) OR TITLE-ABS-KEY ( childbirth ) OR TITLE-ABS-KEY ( "Age at first childbirth" ) OR TITLE-ABS-KEY ( birth ) ) AND ( EXCLUDE ( DOCTYPE , "re" ) OR EXCLUDE ( DOCTYPE , "cp" ) OR EXCLUDE ( DOCTYPE , "ed" ) OR EXCLUDE ( DOCTYPE , "no" ) OR EXCLUDE ( DOCTYPE , "ch" ) OR EXCLUDE ( DOCTYPE , "sh" ) OR EXCLUDE ( DOCTYPE , "le" ) ) AND ( EXCLUDE ( SUBJAREA , "ARTS" ) ) AND ( EXCLUDE ( SRCTYPE , "k" ) OR EXCLUDE ( SRCTYPE , "b" ) )

- **Results:** 259 documents

#2 ( TITLE-ABS-KEY ( "cardiovascular disease" ) OR TITLE-ABS-KEY ( "Myocardial Infarction" ) OR TITLE-ABS-KEY ( "Angina" ) OR TITLE-ABS-KEY ( "Stroke" ) OR TITLE-ABS-KEY ( "Heart attack" ) OR TITLE-ABS-KEY ( "Coronary disease" ) OR TITLE-ABS-KEY ( "Coronary disease" ) OR TITLE-ABS-KEY ( "Cardiovascular mortality" ) OR TITLE-ABS-KEY ( "Acute myocardial syndrome" ) ) AND ( TITLE-ABS-KEY ( menopause ) OR TITLE-ABS-KEY ( postmenopause ) OR TITLE-ABS-KEY ( premenopause ) OR TITLE-ABS-KEY ( "menopausal age" ) OR TITLE-ABS-KEY ( climacteric ) OR TITLE-ABS-KEY ( "Age of menopause" ) OR TITLE-ABS-KEY ( "Age at menopause" ) OR TITLE-ABS-KEY ( menopaus* ) ) AND ( TITLE-ABS-KEY ( abortion ) OR TITLE-ABS-KEY ( "Pregnancy loss" ) OR TITLE-ABS-KEY ( miscarriage ) ) AND ( EXCLUDE ( DOCTYPE , "re" ) OR EXCLUDE ( DOCTYPE , "ed" ) OR EXCLUDE ( DOCTYPE , "cp" ) OR EXCLUDE ( DOCTYPE , "no" ) OR EXCLUDE ( DOCTYPE , "sh" ) OR EXCLUDE ( DOCTYPE , "le" ) ) AND ( EXCLUDE ( SUBJAREA , "CENG" ) OR EXCLUDE ( SUBJAREA , "ENVI" ) )

- **Results:** 57 documents

#3 ( TITLE-ABS-KEY ( "cardiovascular disease" ) OR TITLE-ABS-KEY ( "Myocardial Infarction" ) OR TITLE-ABS-KEY ( "Angina" ) OR TITLE-ABS-KEY ( "Stroke" ) OR TITLE-ABS-KEY ( "Heart attack" ) OR TITLE-ABS-KEY ( "Coronary disease" ) OR TITLE-ABS-KEY ( "Coronary disease" ) OR TITLE-ABS-KEY ( "Cardiovascular mortality" ) OR TITLE-ABS-KEY ( "Acute myocardial syndrome" ) ) AND ( TITLE-ABS-KEY ( menopause ) OR TITLE-ABS-KEY ( postmenopause ) OR TITLE-ABS-KEY ( premenopause ) OR TITLE-ABS-KEY ( "menopausal age" ) OR TITLE-ABS-KEY ( climacteric ) OR TITLE-ABS-KEY ( "Age of menopause" ) OR TITLE-ABS-KEY ( "Age at menopause" ) OR TITLE-ABS-KEY ( menopaus* ) ) AND ( TITLE-ABS-KEY ( "Menstrual Cycle" ) ) AND ( EXCLUDE ( DOCTYPE , "re" ) OR EXCLUDE ( DOCTYPE , "ed" ) OR EXCLUDE ( DOCTYPE , "cp" ) OR EXCLUDE ( DOCTYPE , "le" ) OR EXCLUDE ( DOCTYPE , "sh" ) OR EXCLUDE ( DOCTYPE , "ch" ) OR EXCLUDE ( DOCTYPE , "no" ) OR EXCLUDE ( DOCTYPE , "bk" ) ) AND ( EXCLUDE ( SRCTYPE , "b" ) OR EXCLUDE ( SRCTYPE , "k" ) OR EXCLUDE ( SRCTYPE , "p" ) )

- **Results:** 220 documents

#4 ( TITLE-ABS-KEY ( "cardiovascular disease" ) OR TITLE-ABS-KEY ( "Myocardial Infarction" ) OR TITLE-ABS-KEY ( "Angina" ) OR TITLE-ABS-KEY ( "Stroke" ) OR TITLE-ABS-KEY ( "Heart attack" ) OR TITLE-ABS-KEY ( "Coronary disease" ) OR TITLE-ABS-KEY ( "Coronary disease" ) OR TITLE-ABS-KEY ( "Cardiovascular mortality" ) OR TITLE-ABS-KEY ( "Acute myocardial syndrome" ) ) AND ( TITLE-ABS-KEY ( menopause ) OR TITLE-ABS-KEY ( postmenopause ) OR TITLE-ABS-KEY ( premenopause ) OR TITLE-ABS-KEY ( "menopausal age" ) OR TITLE-ABS-KEY ( climacteric ) OR TITLE-ABS-KEY ( "Age of menopause" ) OR TITLE-ABS-KEY ( "Age at menopause" ) OR TITLE-ABS-KEY ( menopaus* ) ) AND ( TITLE-ABS-KEY ( hysterectomy ) ) AND ( EXCLUDE ( DOCTYPE , "re" ) OR EXCLUDE ( DOCTYPE , "no" ) OR EXCLUDE ( DOCTYPE , "ed" ) OR EXCLUDE ( DOCTYPE , "le" ) OR EXCLUDE ( DOCTYPE , "sh" ) OR EXCLUDE ( DOCTYPE , "cp" ) OR EXCLUDE ( DOCTYPE , "ch" ) ) AND ( EXCLUDE ( SUBJAREA , "CENG" ) OR EXCLUDE ( SUBJAREA , "DENT" ) ) AND ( EXCLUDE ( SRCTYPE , "b" ) OR EXCLUDE ( SRCTYPE , "k" ) )

- **Results:** 316 documents

#5 ( TITLE-ABS-KEY ( "cardiovascular disease" ) OR TITLE-ABS-KEY ( "Myocardial Infarction" ) OR TITLE-ABS-KEY ( "Angina" ) OR TITLE-ABS-KEY ( "Stroke" ) OR TITLE-ABS-KEY ( "Heart attack" ) OR TITLE-ABS-KEY ( "Coronary disease" ) OR TITLE-ABS-KEY ( "Coronary disease" ) OR TITLE-ABS-KEY ( "Cardiovascular mortality" ) OR TITLE-ABS-KEY ( "Acute myocardial syndrome" ) ) AND ( TITLE-ABS-KEY ( menopause ) OR TITLE-ABS-KEY ( postmenopause ) OR TITLE-ABS-KEY ( premenopause ) OR TITLE-ABS-KEY ( "menopausal age" ) OR TITLE-ABS-KEY ( climacteric ) OR TITLE-ABS-KEY ( "Age of menopause" ) OR TITLE-ABS-KEY ( "Age at menopause" ) OR TITLE-ABS-KEY ( menopaus* ) ) AND ( TITLE-ABS-KEY ( "contraceptive agent" ) OR TITLE-ABS-KEY ( "contraceptive device" ) OR TITLE-ABS-KEY ( "oral contraceptive" ) ) AND ( EXCLUDE ( DOCTYPE , "re" ) OR EXCLUDE ( DOCTYPE , "cp" ) OR EXCLUDE ( DOCTYPE , "ed" ) OR EXCLUDE ( DOCTYPE , "no" ) OR EXCLUDE ( DOCTYPE , "sh" ) OR EXCLUDE ( DOCTYPE , "le" ) OR EXCLUDE ( DOCTYPE , "ch" ) ) AND ( EXCLUDE ( SUBJAREA , "SOCI" ) OR EXCLUDE ( SUBJAREA , "CHEM" ) OR EXCLUDE ( SUBJAREA , "AGRI" ) OR EXCLUDE ( SUBJAREA , "ARTS" ) ) AND ( EXCLUDE ( SRCTYPE , "k" ) OR EXCLUDE ( SRCTYPE , "b" ) OR EXCLUDE ( SRCTYPE , "Undefined" ) )

- **Results:** 375 documents
- **Database:** Embase
  - **Date of search:** October, 14 2022
  - **Search strategy with filters:**

#1 (**'cardiovascular disease'**:ab OR **'cardiovascular disease'**:ti OR **'cardiovascular disease'**:kw OR **'heartinfarction'**:ab OR **'heart infarction'**:ti OR **'heart infarction'**:kw OR **'acute myocardial ischemia'**:ab OR**'acute myocardial ischemia'**:ti OR **'acute myocardial ischemia'**:kw OR **'angina'**:ab OR **'angina'**:ti OR**'angina pectoris'**:kw OR **'cerebrovascular accident'**:ab OR **'cerebrovascular accident'**:ti OR**'cerebrovascular accident'**:kw OR **'stroke'**:ab OR **'stroke'**:ti OR **'stroke'**:kw OR **'coronary arterydisease'**:ab OR **'coronary artery disease'**:ti OR **'coronary artery disease'**:kw OR **'coronary disease'**:abOR **'coronary disease'**:ti OR **'coronary disease'**:kw OR **'heart attack'**:ab OR **'heart attack'**:ti OR **'heartattack'**:kw OR **'cardiovascular mortality'**:ab OR **'cardiovascular mortality'**:ti OR **'cardiovascularmortality**:kw) AND ([embase]/lim OR [medline]/lim)

#2 (**'menopause'**:ab OR **'menopause'**:ti OR **'menopaus*'**:kw OR **'postmenopause'**:ab OR **'postmenopause'**:tiOR **'postmenopause'**:kw OR **'premenopause'**:ab OR **'premenopause'**:ti OR **'premenopause'**:kw OR **'climacterium:ab'**OR **'climacterium:ti'**OR **'climacterium:kw'**OR **climacteric**:ab OR **climacteric**:ti OR**climacteric**:kw OR **'menopausal age'**:ab OR **'menopausal age'**:ti OR **'menopausal age'**:kw OR **'age atmenopause'**:ab OR **'age at menopause'**:ti OR **'age at menopause'**:kw OR **'age of menopause'**:ab OR **'ageof menopause'**:ti OR **'age of menopause**:kw) AND ([embase]/lim OR [medline]/lim)

#3 ('parturition':ab OR **'parturition'**:ti OR **'parturition'**:kw OR **'birth'**:ab OR **'birth'**:ti OR **'obstetricdelivery'**:ab OR **'obstetric delivery'**:ti OR **'obstetric delivery'**:kw OR **'delivery'**:ab OR **'age at firstchildbirt**:ab) AND ([embase]/lim OR [medline]/lim)

#4 (**'induced abortion'**:ab OR **'induced abortion'**:ti OR **'induced abortion'**:kw OR **'spontaneous abortion'**:abOR **'spontaneous abortion'**:ti OR **'spontaneous abortion'**:kw OR **'abortion'**:ab OR **'abortion'**:ti OR**'abortion'**:kw OR **'miscarriage'**:ab OR **'miscarriage'**:ti OR **'miscarriage'**:kw OR **'pregnancy loss'**:ab OR**'pregnancy loss'**:ti OR **'pregnancy loss**:kw) AND ([embase]/lim OR [medline]/lim)

#5(**'hysterectomy'**:ab OR **'hysterectomy'**:ti OR **'hysterectomy'**:kw OR **'vaginal hysterectomy**:kw) AND([embase]/lim OR [medline]/lim)

**#6 'menstrual cycle'**:ab OR **'menstrual cycle'**:ti OR **'menstrual cycle**:kw

#7 (**'contraceptive'**:ab OR **'contraceptive'**:ti OR **'contraceptive'**:kw OR **'contracept'**:ab OR **'contracept'**:ti OR**'contracept**:kw) AND ([embase]/lim OR [medline]/lim)

**(#1**AND **#2**AND **#3) OR (#1**AND **#2**AND **#4)** OR **(#1**AND **#2**AND **#5)** OR **(#1**AND **#2**AND **#6)** OR **(#1**AND **#2**AND **#7)** AND ([adult]/lim OR [aged]/lim OR [middle aged]/lim OR [very elderly]/lim OR [young adult]/lim) AND**'article'**/it AND (**'case control study'**/de OR **'clinical study'**/de OR **'clinical trial'**/de OR **'cohortanalysis'**/de OR **'comparative study'**/de OR **'controlled clinical trial'**/de OR **'controlled study'**/de OR**'cross sectional study'**/de OR **'crossover procedure'**/de OR **'feasibility study'**/de OR **'human'**/de OR**'human experiment'**/de OR **'human tissue'**/de OR **'longitudinal study'**/de OR **'major clinical study'**/deOR **'medical record review'**/de OR **'methodology'**/de OR **'multicenter study'**/de OR **'normal human'**/deOR **'observational study'**/de OR **'population based case control study'**/de OR **'proportional hazardsmodel'**/de OR **'prospective study'**/de OR **'randomized controlled trial'**/de OR **'randomized controlledtrial topic'**/de OR **'retrospective study'**/de OR **'secondary analysis**/de OR ‘statistical model’/de) AND([medline]/lim NOT ([embase classic]/lim AND [medline]/lim) OR [preprint]/lim)
