# Supplementary material for: Validation of large language models (Llama 3 and ChatGPT-4o mini) for title and abstract screening in biomedical systematic reviews
Source: Res Synth Methods. 2025 Mar 24;16(4):620–30. doi: 10.1017/rsm.2025.15 (PMC12623132; doi:10.1017/rsm.2025.15)
Supplement: López-Pineda et al. supplementary material [file S1759287925000158sup001.zip › Supplementary_Material_2.docx]

**Supplementary Material 2. Study Selection Criteria**

**Types of Studies**
All clinical trials, cohort studies, and case-control studies that use RR (Relative Risk), OR (Odds Ratio), or HR (Hazard Ratio) as measures of association or effect will be included. Descriptive studies, analytical studies, and clinical trials that use other measures of association or effect will be excluded.

**Study Population (Participants)**
The study population should include women aged 30 to 70 years who are postmenopausal.

**Intervention/Exposure**
This review will include studies that analyze female-specific factors such as age at first childbirth, age at menopause, number of children, number of abortions (spontaneous and induced), menstrual cycle duration, use of contraceptive pills, hysterectomy, hormone replacement therapy, common adverse pregnancy outcomes (APOs) (gestational diabetes, hypertensive disorders of pregnancy, low birth weight, high birth weight, and preterm delivery), and breast arterial calcification. An exposed group with some cardiovascular risk factors specific to menopause and a non-exposed group without risk factors will be considered.

**Outcome Measures**
Any of the following will be considered as outcome measures: 1- Cardiovascular events (including coronary heart disease, fatal and non-fatal acute myocardial infarction, angina pectoris, and stroke) and 2- Cardiovascular mortality.
